# The Multi-Conductivity Clausius–Mossotti Factor as an Electrophysiology Rosetta Stone: Dielectrophoresis, Membrane Potential and Zeta Potential

**DOI:** 10.3390/mi16111200

**Published:** 2025-10-23

**Authors:** Michael Pycraft Hughes

**Affiliations:** Department of Biomedical Engineering and Biotechnology, Khalifa University of Science and Technology, Sheikh Shakhbout bin Sultan Street, Abu Dhabi P.O. Box 127788, United Arab Emirates; michael.hughes@ku.ac.ae

**Keywords:** electrome, electrokinetics, electrorotation, dielectric, membrane potential

## Abstract

Dielectrophoresis (DEP) has been used for decades to estimate the passive electrical properties of cells. However, the body of work on cell electrophysiology derived from Clausius–Mossotti analysis of DEP-derived data pales to insignificance against the wider backdrop of cell electrophysiology based on the Goldman–Hodgkin–Katz equation measured by patch clamp, which focuses on membrane potential *V_m_*—a parameter which does not appear in the Clausius–Mossotti model—and values of patch clamp-derived membrane conductance which, shorn of double-layer conductivity, are often orders of magnitude lower than those derived from DEP. Conversely, the body of work on DEP analysis is more substantial than that reporting the electrical properties of the extracellular (ζ) potential. To address this, several studies have recently been published into the interconnections between the electrical properties determined by the Clausius–Mossotti model, *V_m_*, and ζ-potential, which analyzed the effect of varying the suspending medium conductivity over a wide range, from below 50 mSm^−1^ to above 1.5 Sm^−1^. The results of these studies identified relationships between the cytoplasm conductivity, *V_m_*, membrane conductance and capacitance, surface conductance, whole-cell resistance, and ζ-potential. Significantly, many of these relationships only become apparent when analyzed as a function of the conductivity of the suspending medium. This paper assembles these interconnections, using several separate studies approaching different parameter connections, to draw together a set of equations which collectively form a “cellular electrome”. This demonstrates that analysis of the Clausius–Mossotti factor across multiple conductivities allows determination of not only passive electrical properties, but also the membrane and ζ-potential, and accurately predicts DEP behavior at higher conductivity for the first time.

## 1. Introduction

For over three decades, researchers in cell biophysics have used dielectrophoresis (DEP) and related phenomena such as electrorotation to determine the electrical properties of cells [1]. This is usually performed by analyzing the motion of either single cells or cell ensembles as they interact with an electric field across a range of frequencies. This is then interpreted via the Clausius–Mossotti factor, a mathematical operation which relates DEP behavior to the electrical properties of a particle and medium as a function of frequency [1,2]. From this, researchers then produce values of membrane conductance and capacitance, surface conductance, and cytoplasm conductivity, which are then used to explain biophysical phenomena, such as differences between cell types [3,4,5,6,7,8] or the response of cells to stimuli [9,10,11,12,13]. Those unfamiliar with the principles of DEP can find an introduction to the subject in Appendix A.

However, there have been two issues with the use of DEP in this manner, which have generally received little attention. The first is that it has been known for many years that the ionic strength of the medium in which DEP takes place has a bearing on the measured value of cytoplasm conductivity. This was first reported by Gascoyne and co-workers in 1993 [13] and received more rigorous treatment by Pethig and coworkers in subsequent studies [14,15], but this relationship was not analyzed in detail for many years. The second issue is that whilst DEP produces passive values of resistance and capacitance, it does not produce data one would normally obtain via classical electrophysiology methods such as patch clamp. Whilst the measurement of single ion channel activity would always be beyond the reach of any DEP-based analysis, there are three whole-cell measures that can be determined using patch clamp; whole-cell capacitance and conductance (related to the DEP-derived membrane permittivity and conductivity, as well as the membrane thickness and cell surface area) and cell membrane potential. The last of these is the gold standard of cell electrophysiology yet has no direct measurement in DEP.

A study in 2021 [16] showed significant interactions between multiple bioelectric phenomena including membrane potential, zeta (ζ) potential (the voltage as the hydrodynamic plane of shear, approximately 1 nm outside the cell and related to cell surface charge) [17], surface conductance, membrane conductance, membrane capacitance, and cytoplasm conductivity, as well as the ionic strength of the medium. This led to an exploration of the connection of each of these with the other and has built into a substantial model which is presented here *in toto* for the first time. 

The combination of these parameters, and their interconnections, has been referred to as a *cellular electrome*. The word “electrome” began to gain traction in the early 2010s and first appeared in the published literature in 2017 [18]. That paper did not, however, define what the electrome might be. Subsequent uses have been applied to ionic behavior in plants [19,20,21], and the term recently appeared in the title of a book describing bioelectric phenomena more generally [22]. An electromic approach might be best regarded as taking a holistic approach to the interaction of various cellular phenomena, all of which are rooted in the effects of charge (either fixed on a surface, or mobile charges such as ions) and the potentials and currents these charges cause. The effects of these charges appear in different scientific traditions—electrophysiology, dielectrophoresis, and surface science as well as classical cell biology—but these different manifestations are often caused by the same underlying phenomena, and by understanding these connections we can better understand both the way in which the cell functions electrostatically, and the implications this has for wider cellular function. For this paper (and posterity), the electrome is defined as “the totality of the electrical charges (fixed, mobile, and induced) across a biological system, and the associated electrical potentials, conductances, and capacitances through which they interact. It has implications from the nanoscale (ion channels and double layers) to macro scale (EEG and ECG) and impacts both on how cells function in isolation, how they interact with their environment and other cells, and how ensembles of cells perform multiple biological functions”.

This paper collects recent work uniting a range of electrophysiological parameters to the DEP response, in order to describe the cellular electrome. In particular, it addresses the different ways in which the Clausius–Mossotti factor response changes as a function of medium conductivity, and how these changes can be used to determine other electrical parameters beyond those present in the standard model, creating a “Rosetta stone” connecting DEP, patch clamp, and zeta potential, and allowing determination of membrane and zeta potentials, as well as whole-cell resistance comparable to that obtained by conventional methods. Given the simplicity and rapidity of DEP measurement, this offers a potential new tool for direct measurement of cell electrophysiology.

## 2. Background Theory

### 2.1. An Electromic Rosetta Stone

**Figure 1:**
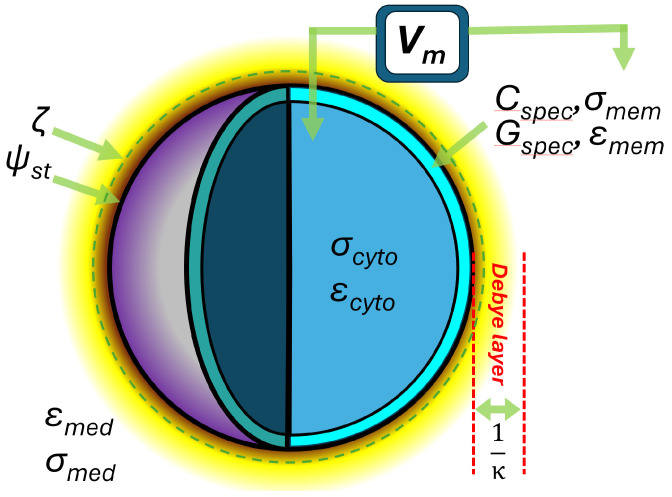
The key elements of the cellular electrome model presented in this paper. The cell surface is characterized by its surface potential *ψ_st_* at the Stern layer boundary and ζ-potential a short distance (ca. 1–2 nm) beyond this; the thickness of the diffuse (Debye) layer of the electrical double layer; the conductivity σ and permittivity ε of the membrane and cytoplasm (subscripts mem and cyto, respectively), with the latter giving rise to the specific membrane capacitance *C_spec_* and conductance *G_spec_*. Finally, the differences in extracellular and intracellular ion content give rise to the membrane potential *V_m_*. Not illustrated: cell radius *r_cell_* and membrane thickness *t*.

The aim of this work has been to unify different schools of thought in cellular bioelectric phenomena—DEP, electrophysiology, and surface science—into a single cellular electrome model, comprising the measures shown schematically in Figure 1. This benefits our understanding of cell function, and also allows one analytical method to be used to measure other electrical phenomena—for example, allowing DEP to estimate the zeta and membrane potentials, by using the Clausius–Mossotti factor as a “Rosetta stone” connecting multiple phenomena. To understand this, we must begin by considering what the different languages of our Rosetta stone might be.

### 2.2. The Clausius–Mossotti Factor

Ottaviano-Fabrizio Mossotti (1791–1863) was an Italian physicist who made a name for himself in Buenos Aires and researched in dielectrics and neuroscience; Rudolf Clausius (1822–1888) was a Prussian physicist whose primary contributions formed the cornerstone of modern thermodynamics. Their roles in the derivation of the expression that bears their name remains something of a matter of contention, as described in Pethig’s excellent historical review [23]. Mossotti’s contribution came in the paper “Analytical discussion on the influence that the action of a dielectric medium has on the surface distribution of electricity of several electric bodies scattered in it” in 1850 [24], refined to its canonical form in Clausius’ book “The Mechanics of Electricity” in 1879 [25]. However, whilst these two names are the ones that we associate with the model, its origins also lie with contemporary British scientists such as Green, Faraday and Maxwell, and French scientists Laplace and Poisson [23]. Nevertheless, the term Clausius–Mossotti factor emerged in the late 1980s and has become the standard terms for the relative polarizability of a suspensoids, at least within the DEP community [23,26].

Mathematical treatments exist for the analysis of ellipsoids (which can be found in Appendix B) [26,27], and indeed, any arbitrary shape [28]. However, most researchers in cell DEP classically approximate the cell to a sphere. For a homogeneous sphere, the DEP force acting upon it is given by:

(1) $$F=2\pi \varepsilon_{med}{r_{cell}}^{3}Re\left[K\left(\omega \right)\right]\nabla E^{2}$$

where *ε_med_* is the absolute permittivity of the suspending medium, *r_cell_* is the radius of the cell, ∇ is the gradient operator, *E* is the magnitude of the electric field, Re denotes the real part, and *K*(ω) is the relative polarizability of the particle with respect to the suspending medium. This is the Clausius–Mossotti factor, given by the expression:

(2) $$K(\omega )=\frac{\varepsilon_{cell}^{*}-\varepsilon_{med}^{*}}{\varepsilon_{cell}^{*}+2\varepsilon_{med}^{*}}$$

where the subscript *cell* refers to the whole cell, *med* to the suspending medium, and ε* denotes complex permittivity, given by

(3) $$\varepsilon^{*}=\varepsilon -j\frac{\sigma }{\omega }$$

where *ε* is the permittivity, *σ* the conductivity, *ω* the angular frequency of the applied field, and *j* the complex operator √−1. Assuming a suspensoid does not change its size or medium composition, the force in Equation (1) is dependent on two things; the local electric field geometry and the value of *K*(*ω*). If the distribution of ∇*E^2^* remains constant—that is, the RMS electric field geometry remains fixed within the analysis volume [29]—then the DEP force is dependent only on constants (2, π, *ε_med_*, *r_cell_*, ∇*E^2^*) and on *K*(*ω*). Consequently, analysis of variation in DEP force as a function of frequency yields a spectrum that is dependent on constants and the value of Re[*K*(*ω*)] at each tested frequency. This means that the particle properties can be determined where the properties of the medium are known, by fitting the DEP spectrum from Equation (2), multiplied by a scaling factor.

**Figure 2:**
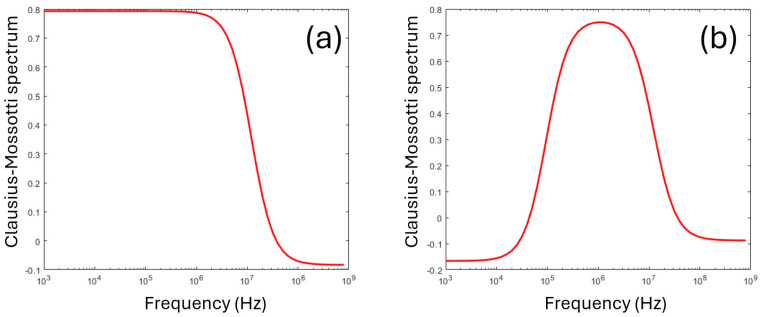
Typical Clausius–Mossotti spectra for (**a**) a homogeneous sphere, per Equation (2), and (**b**) a shelled sphere, with a thin membrane surrounding a conducting core, per Equation (4). The latter model is commonly used for modeling cells.

Equation (2) describes a single transition (known as a dielectric dispersion) from the steady-state value at low frequencies, to the steady-state value at high frequencies. The frequency where *K*(*ω*) = 0 as it crosses over from positive to negative (termed the crossover frequency *f_x_*) has been used as a means of determining the dielectric parameters [1] with the use of suitable approximations (since only one parameter is measured, it is impossible to determine both permittivity and conductivity and usually only the former is measured). A typical dispersion following Equation (2) can be seen in Figure 2a.

For more complex structures such as cells, an extension to the Clausius–Mossotti factor allows a more complete understanding of the makeup of the cell. This works by considering a sequence of nested shells surrounding a central core. The process begins with the innermost core and the first shell, where the core is envisaged suspended in a medium comprising the shell; the net result is then imagined nestling within a medium comprising the next shell, and onwards until all shells and the medium itself are included. Whilst some practitioners have used multi-shelled models to predict the dielectric properties of up to four cell components (nucleus, nuclear membrane, cytoplasm, and cell membrane), there are too few unique features of the Clausius–Mossotti spectrum to determine so many unique parameters. In general, the spectrum is reduced to four key points; the starting value of the spectrum at low frequency, the end value at high frequency, and the frequencies of the two dielectric dispersions; the first of which causes the spectrum to rise with increasing frequency, and the second causing it to fall at high frequencies. To achieve this, we need to substitute for *ε_cell_* in Equation (2), using the following expression [1,2,30,31]:

(4) $$\varepsilon_{cell}^{*}=\varepsilon_{m}^{*}\frac{{\left(\frac{r_{cell}}{r_{cell}\;-t}\right)}^{3}+2\frac{\varepsilon_{cyto}^{*}-\varepsilon_{mem}^{*}}{\varepsilon_{cyto}^{*}+2\varepsilon_{mem}^{*}}}{{\left(\frac{r_{cell}}{r_{cell}-t}\right)}^{3}-\frac{\varepsilon_{cyto}^{*}-\varepsilon_{mem}^{*}}{\varepsilon_{cyto}^{*}+2\varepsilon_{mem}^{*}}}$$

where *t* is the membrane thickness, and subscripts *_cyto_* and *_mem_* refer to the properties of the cytoplasm and membrane, respectively. The combined values of Equations (2) and (4) yield a spectrum of Re[*K*(*ω*)] such as the one in Figure 2b.

Whilst Equation (4) yields the membrane conductivity and permittivity, accurate determination of both values is dependent on accurate measurement of the membrane thickness *t*, which is very difficult to measure (requiring electron microscopy or better). Consequently, it is usual to assume a value, with 7 nm being common.

The values of membrane permittivity and conductivity as derived from Equation (4) both scale with *t*; using a value of *t* of 14 nm yields values of membrane permittivity and conductivity that are twice as large as those determined where *t* = 7 nm, for the same spectrum. We can eliminate this dependence by dividing the derived parameters by *t*, which yields the specific membrane capacitance (that is, capacitance per unit area) *C_spec_* (=*ε_mem_*/*t*) and specific membrane conductance (conductance per unit area) *G_spec_* (=*σ_mem_*/*t*). These two values are sometimes referred to as the effective specific membrane capacitance *C_eff_* and conductance *G_eff_*.

These represent values per unit area of membrane as determined from Equation (4), which assumes a spherical cell with a smooth membrane. We can then use the area of that sphere (that is, 4π*r_cell_*^2^ where *r_cell_* is the radius used in Equation (4)) to produce values of whole cell capacitance *C_wc_* and conductance *G_wc_*. Measured values of *C_cw_* have been shown to be similar to those measured using patch-clamp [32,33]; the value of *G_spec_* is however different to that derived from whole cell resistance *R_wc_*. The reasons for this are discussed in Section 4.4 and Section 4.5.

### 2.3. Membrane Potential

To compare DEP-derived electrophysiology data to other, more common electrophysiological methods, it is important to consider the phenomena they study. The most commonly used whole-cell electrophysiological measure is the cell membrane potential *V_m_*, which typically takes a range of values from around 0 mV to −100 mV in mammalian cells, with many (particularly the most-studied, “excitable” cells of muscle and nerve tissue that employ *V_m_* to function) in the −70 mV to −85 mV range. Determination of the membrane potential was a focus of electrophysiology throughout the nineteenth century, with the role of electricity in biological function having been identified by Galvani in the late 1700s [34]. It was in the 1800s that Emile duBois Reymond [35] identified the origin of *V_m_* as arising from the partition of ion species across the cell membrane; the cytoplasm has much higher levels of potassium (K^+^) and lower levels of sodium (Na^+^) compared to the extracellular fluid, and these electrochemical gradients drive the membrane potential [36]. Since the process is ultimately diffusive, the expression for *V_m_* derived by Julius Bernstein [37] drew heavily on the general work of Nernst on diffusion [38], which led to the equation bearing Nernst’s name rather than Bernstein’s. The expression for a monovalent cation is as follows:

(5) $$V_{m}=\frac{RT}{F}ln\left(\frac{c_{out}}{c_{in}}\right)$$

where *c_out_* and *c_in_* describe the external and internal ion concentrations, and *R*, *T*, and *F* have their usual thermodynamic meanings. The denominator and numerator in the logarithm term are exchanged in the case of an anion being dominant. In the case of most cells, the dominant ion was believed to be K^+^ and the expression was generally written with K^+^_in_ and K^+^_out_ to reflect this. However, by the 1940s, with better instrumentation and models built around the physically large giant squid axon, it became apparent that other ions also played a role [39,40,41]. This led to the extended version of Equation (5) for the three primary monovalent ions, K^+^, Na^+^, and Cl^−^ (chloride) posited by David Goldman in his famous “constant field” model [42], later adapted by Hodgkin and Katz to describe *V_m_* in muscle tissue [43] in the now-canonical form of the equation (for this reason, it is referred to either as the Goldman equation, or the Goldman–Hodgkin–Katz (GHK) equation). The model considers membrane potential *V_m_* creating a uniform electric field within the cell membrane, with zero electric field outside it. The GHK equation is typically written as follows:

(6) $$V_{m}=\frac{\mathrm{RT}}{F}\ln\left(\frac{P_{{Na}^{+}}{\left[{Na}^{+}\right]}_{out}+P_{K^{+}}{\left[K^{+}\right]}_{out}+P_{{Cl}^{-}}{\left[{Cl}^{-}\right]}_{in}}{P_{{Na}^{+}}{\left[{Na}^{+}\right]}_{in}+P_{K^{+}}{\left[K^{+}\right]}_{in}+P_{{Cl}^{-}}{\left[{Cl}^{-}\right]}_{out}}\right)$$

where *P_x_* is the ion permeability for ion *x* (either Na^+^, Cl^−^, or K^+^). If conduction is dominated by one ion, then Equations (5) and (6) are similar.

### 2.4. Zeta Potential (ζ)

Another electrical measurement which has been applied to cells—albeit far less frequently than measurement of *V_m_* or DEP—is the zeta potential (ζ). ζ is the electrical potential arising due to the charges on the surface of any object in an aqueous solvent [44]. To understand this, we must consider the complex situation arising at the interface between a charges surface and a suspending medium. This complexity arises because there are two processes which occur in parallel at this interface: an electrical process and a hydrodynamic process. Let us consider these separately.

In electrical terms, the charge on the surface attracts countercharge (either counterions or the charged ends of polar molecules) and repels coions with like charge. This results in a layer of charge around the cell in which the ion concentrations deviate from the bulk; this is known as the *electrical double layer*. Immediately outside the cell surface, some counterions will be electrostatically bound to the surface, and immobilized; as will the positive ends of some water dipoles. These form a thin aqueous shell around the surface called the Stern layer, which terminates at the Helmholtz plane. Beyond this is a second zone (the Debye layer) where the medium ions are free to move but vary in concentration from the bulk with elevated counterions and reduced coions. The concentration of any ion *c_i_*(0) with valence *z* deviates from the bulk ion concentration *c_b_* according to the Poisson–Boltzmann equation:

(7) $$c_{i}\left(0\right)=c_{b}exp\left(\frac{{-z}_{i}e\Psi_{s,o}}{kT}\right)$$

where *Ψ_s,o_* is the potential at the surface of the outer membrane surface, and *e*, *k*, and *T* have their usual thermodynamic meanings. The boundary for the electrical double layer is generally defined as the Debye length κ^−1^, given by the expression:

(8) $$1/\kappa =\sqrt{\left(\frac{\varepsilon RT}{2czF^{2}}\right)}$$

where *c* is the electrolyte concentration (mol m^−3^). The changes in ion concentration described by Equation (8) gives rise to an electrical potential *ψ* that begins at the Helmholtz plane with value *ψ_st_* and reduces with distance *r*, thus:

(9) $$\psi (r)=\psi s_{t}\;e^{-(\mathrm{kr})}$$

In parallel to the electrical structure of the extracellular space, there is also the second, hydrodynamic structure. It is a principle of fluid dynamics that fluids are immobile when in contact with solids and move at the same speed as the surface; that is, at the point of interface the fluid is stagnant, and only moves freely a few molecules beyond the interface [45]. The plane delineating the stagnant layer from free-moving solution is referred to as the “hydrodynamic plane of shear”, or the “slip plane”. This is independent of the electrical structure, and the slip plane is usually found beyond the Helmholtz plane, within the Debye layer. The ζ potential is the value of *ψ* at the location of the hydrodynamic plane of shear, typically 0.5–2 nm from the surface of the cell. It is worth noting that whilst the Debye length of Equation (8) depends on medium ion composition, the location of the slip plane does not. Consequently, increasing the ionic content of the medium reduces the Debye length, and compresses the exponential describing the potential from Equation (9) into a shorter distance. Since the slip plane is a fixed distance from the surface, this means a raised medium conductivity will lead to a reduced value of ζ.

ζ is a common measurement in materials science, colloid chemistry, and chemical engineering. It is used to measure the stability of solutions; that is, in determining whether suspended particles have enough charge to repel each other against the attractive van der Waals force. Typically, if a solution contains particles with |ζ| > |±10| mV the solution will be stable; if |ζ| < |±5| mV, it will tend to flocculate, and the suspensoids will clump together. The classical example of this is milk, which contains billions of lipid droplets (micelles), whose ζ = −17.5 mV; adding lemon juice to milk moves the micelles towards their isoelectric point, reducing ζ to −3.5 mV and causing the milk to curdle [46].

ζ is infrequently studied in cells, since the membrane composition (and hence surface charge) is unlikely to change significantly, leading to the misapprehension that it is unlikely to be significantly different from one cell type to the next. However, a comprehensive study of published values of ζ in cells [17] suggested that cells manifest a range of values, from −10 mV for platelets and red blood cells to −31 mV for metastatic breast cancer. It has been shown that cancerous cells are more depolarized than their healthy counterparts, and that macrophages and platelets depolarize on activation. Bacteria occupy an even wider range from +14 mV to −49 mV depending on the strain and conditions, with it being noted that different values of ζ corresponded to different bacterial behavior—whether monodisperse, clustered, or filamentous [17].

## 3. The Cellular Electrome Model

The Hungarian physicist, Nobel prizewinner, and proponent of bioelectric research Albert Szent-Gyorgyi kept a shark hook in his office as a reminder that, to catch a big fish, one needs a big hook; and to catch a big answer, one needs a big question [47]. The big question posited here is “can DEP be used to determine other electrophysiological parameters such as *V_m_*, ζ, *C_wc_*, and *R_wc_*?” To address this, a substantial data set was assembled, initially around a single cell type; the erythrocyte or red blood cell (RBC). RBCs have many advantages: they are highly regular in size and shape; they are well-characterized by a variety of electrical methods; lacking gene expression and mitochondria, they are simple and are amenable to unusual methods of direct measurement of membrane potential; and they are plentiful. RBCs had also previously demonstrated interesting behavior, such as circadian rhythms exhibiting contrapuntal circadian rhythms in *G_spec_* and *σ_cyto_* [33,48]; indeed, it was the synchronicity between these two parameters, and the extent of *G_spec_* movement that would be beyond the explanation of simple channel activity, that in part inspired the initial development of this model. It was also known that *G_spec_* or *C_spec_* (or, possibly, both) exhibited an unexpectedly strong dependence on *σ_med_* [49]. The data set comprised DEP data (*C_spec_*, *G_spec_*, *σ_cyto_*), ζ-potential, and *V_m_* in three conductivities ranging from 17 mSm^−1^ to 1.7 Sm^−1^, and with four chemical treatments (DMSO, DMSO plus valinomycin; neuraminidase; and DMSO plus valinomycin and neuraminidase). Subsequently, other cell types were added to this, providing additional data for platelets, neurons, monocytes, cancer cells, stem cells, and so on. It is also to be noted that an advantage of these studies was that they were performed using measurement apparatus that were able to take measurements to such high conductivities [32]. Most DEP experiments are performed using planar thin-film gold electrodes, which are unable to sustain substantial currents and which often electrolyze at medium conductivities much above 0.1 Sm^−1^. Conversely, the data set here was taken using a platform using much thicker copper electrodes [32], which allowed measurements at conductivities up to 1.7 Sm^−1^, the first time cell DEP spectra had been measured at such conductivities. The ability to measure across a much wider range of conductivities than before was instrumental in the development of the electrome model.

The key findings of this work are expanded upon in the following sections: the most important discovery was that DEP parameters, far from remaining static across media of different conductivities, change in ways that yield valuable electrophysiological data. It is these gradients that form the interconnection between the different electrophysiological forms.

As a starting point, we can consider a connection between two potentials associated at the cellular (rather than organelle) level: the membrane potential *V_m_* and the zeta potential ζ. Notionally, these two are functionally separate; one arises purely because of ion compartmentalization and diffusion, whilst the other arises purely from static charges on the cell surface. As such, they should be unconnected and independent; however, it has been known since the 1970s that this is not the case, where cancer cells [50], mitochondria [51], and slime molds [52] were shown to exhibit a relationship between *V_m_* and ζ. This was later elaborated by Voight and Donath [53], who hypothesized the relationship arose from capacitive division of *V_m_* across membrane and double layers, and described an equation for the contribution of *V_m_* to ζ, referred to here as Δζ, thus:

(10) $$\zeta =\zeta^{\prime }+\Delta \zeta ;\Delta \zeta =\frac{V_{m}C_{mem}}{\left(C_{mem}+C_{dl}\right)}$$

where ζ′ is the zeta potential due solely to the surface chemistry, and *C_dl_* is the double layer capacitance. This was later refined analytically by Dukhin in 1990, who considered the underlying mechanisms and extended the equation to account for contributions from charged surfaces [54,55]. However, this work was circulated primarily among the colloid science community, and despite the potentially significant impact this may have on cell biology, it never reached that audience.

The work was largely forgotten, until the phenomenon was rediscovered in 2021 [16]. In that work, Hughes and colleagues described the additional component of ζ as *ΞV_m_*, where Ξ was best fit by the empirical expression

(11) $$\zeta =\zeta^{\prime }+\Xi V_{m};\;\Xi =\frac{6.7}{1+C_{shear}^{2}}$$

where *C_shear_* is the capacitance of the part of the double-layer between the hydrodynamic plane of shear and the cell surface. Significantly, the values of Ξ observed in cells are substantially larger than might be expected where only the capacitances of the membrane (assumed around 10 mFm^−2^) and double layer (much closer to 1 Fm^−2^) are considered, which would suggest that Δζ would be less than 0.01. Instead, values have been measured between around 0.2 in cardiomyocytes [56] and platelets [57] to around 0.37 in red blood cells [16] and algae [58].

The implications for this interdependence are wide-ranging. In both models, the capacitance of the double layer is key, suggesting that the membrane potential *V_m_* is dropped across a capacitive potential divider that encompasses both the membrane and the electrical double layer, and suggests that the model of ζ, *V_m_*, and passive properties of conductance and capacitance form an inter-related whole with significant implications for cell behavior. The finding also demonstrated a new use of measurement of ζ as a marker for *V_m_*. This was demonstrated by Chacar et al. [56] who used an off-the-shelf ζ-potential instrument to measure *V_m_* of cardiomyocytes in polarized (*V_m_* = −85 mV), depolarized (*V_m_*= +20 mV), and permeabilized (*V_m_* = 0 mV) states and demonstrated that ζ offers a new method of performing cell electrophysiology. Work on platelets where ζ-potential and *V_m_* were measured, as well as DEP parameters and antibody binding [57] showed that modulation of *V_m_* altered both ζ-potential and antibody binding, suggesting that cells can mechanistically alter extracellular interactions by altering *V_m_*. A similar effect was observed in red blood cells [16] where alteration of ζ by altering *V_m_* on the extracellular ion concentration has potential to confer *V_m_*-gating on ion channels without the requirement for a molecular mechanism. In the platelet study, notable connections were observed between electrical properties related to the cell interior (*σ_c_*, *V_m_*) and exterior (*G_spec_*, ζ-potential, antibody binding); in particular, connections were observed between internal and external properties under all circumstances, whereas relationships between internal and external properties were only observed under conditions where Ξ was stable [57]. This leads to the crux of this paper; the intersection of Clausius–Mossotti-derived parameters and their interactions with each other, the medium, and the associated cellular potentials.

## 4. The *σ_med_*-Dependent Clausius–Mossotti Response

### 4.1. The Standard Clausius–Mossotti Interpretation

**Figure 3:**
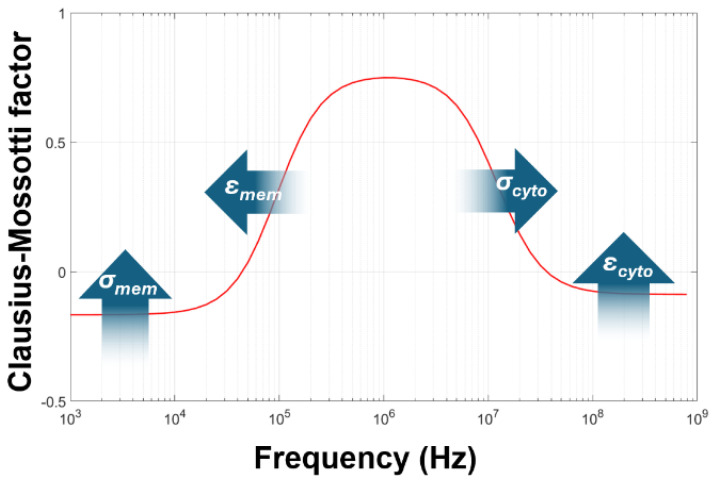
The standard method of modeling the Clausius–Mossotti spectrum for cells of known radius. Fits to the data can be found by altering the four parameters in the figure; the arrows indicate the effect of increasing that parameter.

As discussed in Section 1, the Clausius–Mossotti model has been used for determining the passive electrical properties of cells for three decades. This is performed by measuring the DEP response over a frequency range (typically 10 kHz to 10 MHz, with some researchers extending this up or down by a decade or more), and then fitting Equation (4) to the DEP spectrum by varying *σ_cyto_*, *σ_mem_*, *ε_cyto_*, and *ε_mem_* to fit for a given value of medium conductivity *σ_med_* (the permittivity *ε_med_* of the medium is generally fixed at 78·ε0 or 80·ε0, though in reality the sugar content may make this slightly lower) [59]. Analysis of Equation (4), as summarized in Figure 3, showed that the low-frequency, starting plateau value is mostly governed by the membrane conductivity *σ_mem_*; the frequency of the low-frequency dispersion is governed by the membrane permittivity *ε_mem_*; the frequency of the high-frequency dispersion is governed by cytoplasm conductivity *σ_cyto_*; and the high-frequency plateau (often reached at frequencies beyond the limit of most signal generators used in DEP analysis, making accurate evaluation difficult) is governed by *ε_cyto_* [29]. For the three parameters that were determined most easily, narratives were provided that described what the values signified. First, the membrane conductance primarily describes the ion transport through the membrane (the transmembrane conductance), though an additional term was identified suggesting an effect through the electrical double layer (the tangential membrane conductance) was present in some cells [60]. However, comparison to patch clamp-acquired data did show some close comparisons for some cells between DEP-derived membrane conductance and those derived from canonical methods [61]. Second, the membrane capacitance was assumed to be a measure of membrane folding, since the membrane is primarily composed of a lipid bilayer which has a defined capacitance per unit area when stretched flat [62]. Finally, the cytoplasm conductivity was assumed to represent the quantity of free ions in the bulk cytoplasm. Importantly, it was generally assumed that all three parameters should be constant, homogeneous, and unaffected by medium composition; specifically, that the membrane properties should not be affected by extracellular ion concentration (since the system is regarded as electrically passive, effectively a resistor and capacitor in parallel), though observations of red blood cells in media of different conductivity had shown changes that could be attributed to either a change on membrane conductance or capacitance as a function of *σ_med_* [49].

Similarly, the intracellular ion concentration was assumed to be retained from its state in culture, leading for example to a widely held belief that all cells exhibit only negative DEP at higher conductivities. This assumption of an unchanging cytoplasm has been challenged more frequently; it had been known for several years that the medium conductivity can play a significant role in the measurement of cytoplasm conductivity. For example, Gascoyne and colleagues observed changes in Friend murine erythroleukaemic cells after resuspension in low-conductivity medium [13] which they attributed to cell leakage. A more detailed study by Chung et al. [14,15] showed again a dependence on *σ_med_* that led to a reduction in the high-frequency crossover, and hence to the measurement of *σ_cyto_*. In both cases, the effect was attributed to ion leakage from cells leading to an anomalous value of *σ_cyto_*. Implicit in this analysis is the idea that cytoplasm conductivity should be independent of *σ_med_*; that the value measured by DEP should reflect the value of *σ_cyto_* in normal physiological conditions; and that leak represents a deviation from that ideal state, producing results that are potentially misleading and likely meaningless. The only paper [63] to identify a pattern in such behavior was taken by a related technique (electrorotation), which suggested that not only did varying *σ_med_* lead to a change in *σ_cyto_*, but that the change might be linear.

### 4.2. σ_cyto_ Varies Linearly with σ_med_

**Figure 4:**
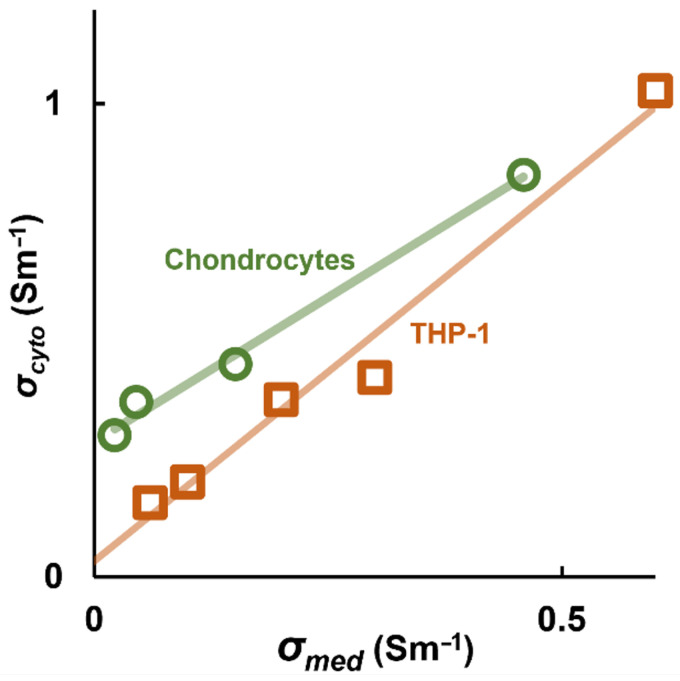
The measured value of *σ_cyto_* from the Clausius–Mossotti factor scales linearly with medium conductivity *σ_med_*, as illustrated in here with two example cell types, the THP-1 monocyte line and primary bovine chondrocytes. Data originally published by Hughes et al. [63].

Following the work of Sukhorukov and colleagues [63], analysis of several cell types demonstrated that *σ_cyto_* consistently varies as a function of *σ_med_* in a linear manner. Analysis of the RBC data showed a linear relationship (R^2^ > 0.99) across the five treatment conditions [16]; subsequent analysis has shown similar relationships in chondrocytes and monocytes (both shown in Figure 4), as well as platelets and cancer cells [63] in addition to the Jurkat data obtained by electrorotation [63]. This was modeled using the classical equation of a straight line, *σ_cyto_* = *A· σ_med_* + *B*, where value *B* represents the value of cytoplasm conductivity independent of the medium, and which may represent the contribution of charges within the cytoplasm that are unable to equilibrate across the membrane, such as charged cytosolic macromolecules. The value of *B* varies between cell types, with estimates varying from 96 mSm^−1^ in RBCs to 300 mSm^−1^ in chondrocytes. HeLa and Jurkat cells were found to have *B* values near to zero.

The gradient value *A* was observed to change between cell types. By analyzing *A* in RBCs, Hughes et al. [64] suggested that *σ_cyto_* does not reflect the average ion concentration (and hence conductivity) of the cytoplasm, but instead reflects the conductivity at the interface between the cytoplasm and the inner membrane surface, in the inner surface’s electrical double layer. They found that *A* could be approximated with the equation

(12) $$A=\frac{2}{exp\left(\frac{-ze({2\Psi }_{sto}-\Psi_{sti})}{kT}\right)+exp\left(\frac{ze({2\Psi }_{sto}{-\Psi }_{sti})}{kT}\right)}$$

where *ψ_sti_* and *ψ_sto_* represent the surface potentials on the inner and outer surfaces of the membrane, respectively. This is because, if we consider cytoplasm content to be equilibration due to simple diffusion, we expect the ion concentrations in the cytoplasm to be the same as those in the bulk. However, the ion concentration at the outer surface—representing the ions available for diffusion across the membrane—is not the same as the bulk; it is altered by the Poisson–Boltzmann equation (Equation (7)), which (for a negatively charged surface) elevates cation concentrations and suppresses anion concentrations. This is reflected in the two exponential terms in the denominator (one for cations and one for anions), which differ from the ion concentration in the bulk (the 2 in the numerator, representing the anions plus cations). However, if the cytoplasm reflects the ion concentration in the outer double layer, then this will be further altered as the ion concentrations then interact with the inner membrane surface, which alters the ion concentration further. Since the Clausius–Mossotti factor is about interfacial relationships, it is the charge concentration at this interface which we most likely measure when we determine *σ_cyto_* from Equation (2)—*not* the ion concentration in the bulk cytoplasm.

### 4.3. V_m_ Depends on σ_cyto_ and σ_med_

Among the electrophysiological parameters that can be obtained by DEP, the most conspicuous absence is *V_m_*, the primary electrophysiological parameter derived from whole-cell patch clamp and many other methods. Previous correlations had been made between *σ_cyto_* and *V_m_* [65]; this is logical, since both relate to the ion concentration in the cytoplasm. However, there was no direct analytical link between them.

This was first addressed using the large RBC electrome study, in which measurements of *V_m_* and *σ_cyto_* were made over a range of conductivities and chemical treatments. This yielded the surprising result that the two scaled linearly with medium concentration—and that the difference between the different conditions could be accounted for by parameter *A*, the slope of *σ_cyto_* as a function of *σ_med_* from Equation (10). This data set demonstrated that *V_m_* in RBCs in media of low conductivity varies substantially—something which had been demonstrated previously using the carbonylcyanide-m-chlorophenylhydrazone (CCCP) method of measuring pH following treatment with a proton ionophore [66]; this cannot be obtained directly by patch clamp, which requires cells to be in high-conductivity physiological medium to function. If we examine the simplest conventional model of membrane potential, the Nernst equation (Equation (5)), we see that there is an inbuilt ratio of external and internal ions. If we consider that a change in extracellular ion concentration δ*c_out_* causes a commensurate change in intracellular ion concentration δ*c_in_*, then substituting these into Equation (5) suggests that this ratio may produce a value of *V_m_*, assuming that *V_m_* remains constant across that range of conductivities (as has been observed in neurons, for example [43]). Indeed, the equation would also suggest that if *V_m_* remains constant over a range of extracellular ion concentrations, then cytoplasm conductivity would be *expected* to change.

**Figure 5:**
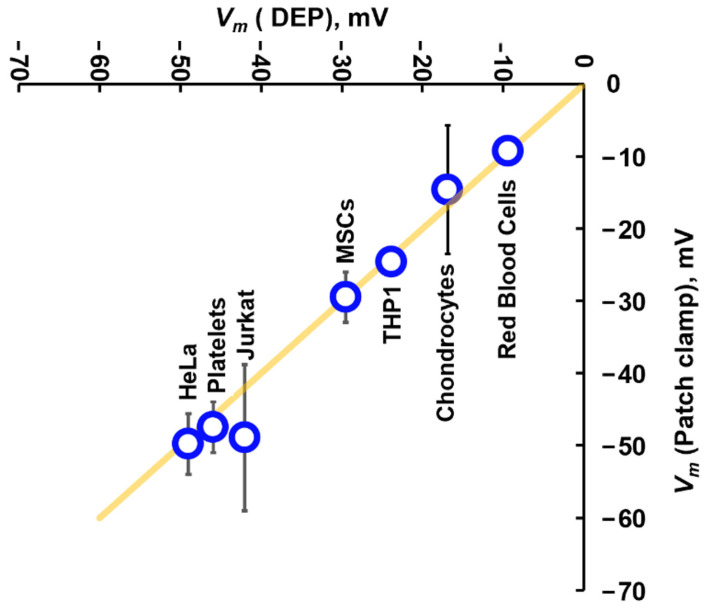
A comparison of *V_m_* values calculated using Equation (13) for a range of cells, plotted against the mean of published values of *V_m_* for the same cell types when measured using patch clamp. The yellow line indicates the line of identity, where DEP and patch clamp yield the same result. Data originally presented by Hughes et al. [63].

This model was tested in a second study [67] which included many other cell types including HeLa cancer cells, chondrocytes, and THP-1 monocytes (examples of which can be seen in Figure 4), as well as the Jurkat data published by Sukhorukov et al. [63]. This data set used DEP to measure *σ_cyto_* at multiple conductivities, and compared the derived slope of change in cytoplasm conductivity *σ_cyto_* for a given change in medium conductivity *σ_med_*. When this was compared to the literature values of *V_m_* acquired in physiological media (about 1.8 Sm^−1^) determined with patch clamp, a relationship was determined between that slope and *V_m_*. The two data sets led to the development of two models, with different purposes. This larger data set led to an equation, adapted from Equation (5), which delivers a highly accurate (typically to within 1–2 mV) estimation of the values of published *V_m_* measured in physiological media, as taken by patch clamp. The DEP data are of course not taken at the same conductivity that the patch clamp data were measured at (DEP data were typically taken at conductivities below 500 mSm^−1^) yet yielded accurate predictions of patch clamp-measured values. The expression was based on the Nernst equation (Equation (5)), but substituting ion concentrations with derived conductivity values [67]:

(13) $$V_{m}=-\frac{RT}{F}\left|ln\left(\frac{{\delta \sigma }_{med}\;}{{\delta \sigma }_{cyto}}\right)\right|+\Psi_{sto}\;=\;-\frac{RT}{F}\left|ln\left(A\right)\right|+\Psi_{offset}$$

where *Ψ_offset_* was found to be a constant −12 mV for all the cell types examined (RBCs, monocytes, platelets, cervical cancer, leukemia, stem cells, primary chondrocytes). As shown in Figure 5, this yielded estimates of *V_m_* very similar to measurements provided by patch clamp. The equation was found to work for all cell types examined, and represents the key Rosetta stone translation between DEP and conventional electrophysiology. The −12 mV offset was found to be universal across all cell types, and may represent the surface potentials of the two cell surfaces (which are predominately lipid in origin, and do not vary much between cell types). The model predicts that *V_m_* is largely uniform across different medium conductivities, in line with observations of squid axons [43].

However, RBCs are unusual, in that studies have shown that *V_m_* is highly affected by medium conductivity, becoming positive at low ion concentrations when measured using the CCCP method [66]. When investigated using CCCP in parallel with DEP, *V_m_* of RBCs was found to typically be around +26 mV in 16 mSm^−1^ solution, and around +20 mV in ca. 160 mSm^−1^ solution [16]. A model was developed based on the capacitance between cytoplasm and medium, which suggested that an excess of cations on the inner leaflet of the membrane, with the charge neutrality in the bulk acting as a 0 V reference, acts as a charged capacitor:

(14) Vm= 1κσmedΛeA2C+Vx

where *C* is the capacitance of the membrane and associated electrical double layers, *e* is the electronic charge, and *Λ* is the number of ions per cubic meter for a conductivity of 1 Sm^−1^. *V_x_* was a constant which held values between +21 mV and +27.5 mV. This model is different to the general model of Equation (13), though both yield similar results at physiological conditions. The difference is that whilst Equation (14) is proportional to medium conductivity, Equation (13) is not. One way that they can be reconciled is by altering the value of *Ψ_offset_* in Equation (13) to account for different media; for RBCs, Equation (13) yields similar results to Equation (14) if this is changed to +28 mV for media with conductivity 160 mSm^−1^ and +35 mV for conductivity 16 mSm^−1^. The physics underpinning this offset is as yet unresolved; for those wishing to explore this, these three offsets are given by either of these empirical expressions:

(15) $$\Psi_{offset}=-\frac{\sigma_{med}}{42.2}+0.02\;\mathrm{or}\;\frac{RT}{F}\left({1-\sigma }_{med}\right)$$

Whether this effect is observed solely in RBCs or is present in other cells, we are unlikely to determine due to the difficulties of direct measurement on *V_m_* in low-salt media. However, −12 mV remains consistent for all cell types in Equation (13), which in light of the finding about *V_m_* in neurons [43] remaining stable across a range of values, suggests this may be an RBC-specific phenomenon.

### 4.4. G_spec_ Depends on V_m_, ζ, and σ_cyto_

As described earlier, one of the reasons for the selection of RBCs as a model for electrome study was prior observation of unusual behavior in *G_spec_*; Gascoyne and coworkers [49] noted that it increased significantly with *σ_med_*, an observation confirmed by Hughes et al. in 2021 [16]. The other unusual measurement was the observation of circadian rhythms in RBCs, where rhythmic behavior in *G_spec_* and *σ_cyto_* was in antiphase. This was attributed to the membrane conductance rising due to active channel pumping to reduce the cytoplasm content, though comparison with patch clamp data suggests that the amplitude of *G_spec_* was much higher than might be expected from channel activity.

The conductance of the membrane as determined by DEP has long since been known to contain both transmembrane (along the bulk-to-cytoplasm axis) and tangential (conduction around the cell, through the electrical double layer) components [60]. This has been explored most deeply in the behavior of latex nanoparticles, which (due to their size, and being nonconductive) are far more dependent on tangential conduction as the primary source of conduction.

**Figure 6:**
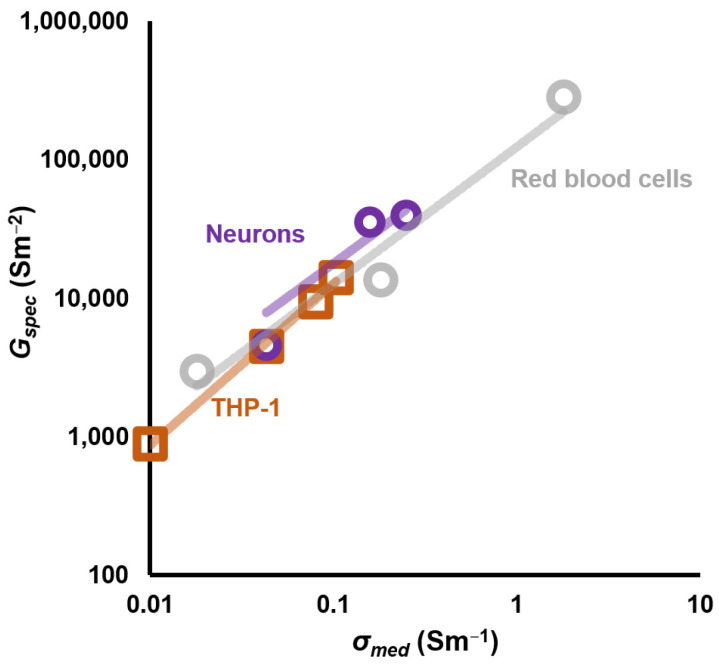
A plot of the DEP-derived values of specific membrane conductance *G_spec_* for a range of values of medium conductivity *σ_med_*, together with best-fit lines according to Equation (19). Data from Hughes et al., [68,69].

Based on existing models of nanoparticles, the value of *G_spec_* was anticipated to be

(16) $$G_{spec}=\frac{1}{t}\left(\sigma_{mem}+\frac{2K_{s}}{r_{cell}}\right)$$

where the *σ_mem_* component represents the transmembrane contribution and the *K_s_* component represents the tangential contribution. The latter is well-characterized from colloid science [44] via the Bikerman equation:

(17) $$K_{s}=\frac{\left(4F^{2}cz^{2}D(1+3m/z^{2})\right)}{RT\kappa }\left(\cos h\left[\frac{zq\zeta }{2kT}\right]-1\right)$$

where *R*, *T,* and *F* are as before, *z* the valence of the counterion, *D^d^* is the ion diffusion coefficient in the diffuse layer, ζ is the ζ-potential, and *m* is given by:

(18) $$m={\left(\frac{RT}{F}\right)}^{2}\frac{2\varepsilon_{m}}{3\eta D^{d}}$$

and where *η* is the viscosity. However, this did not fit the observed behavior in RBCs or other cells (such as shown in Figure 6)—in particular, the dependence of observed values of *G_spec_* at higher conductivities, which have a strong *σ_med_* correlation. Empirical fitting to data [68] revealed that an excellent fit was achieved when:

(19) $$G_{spec}=\frac{\sigma_{med}}{2r_{cell}}-\frac{K_{s}}{2tr_{cell}}$$

where the expression for *K_s_* was adapted from Equation (8), replacing ζ with the Stern layer potential *Ψ_sto_* and *Ξ V_m_*, where *V_m_* is the membrane potential and Ξ represents the proportion of *V_m_* observed at the shear plane, as described previously [16]:

(20) $$K_{s}=\frac{\left(4F^{2}cz^{2}D^{d}(1+3m/z^{2})\right)}{RT\kappa }\left(\cosh\left[\frac{zq\left(\psi_{st}+\Xi V_{m}\right)}{2kT}\right]-1\right)$$

Subsequent work has shown that this model also fits many other cell types [69].

This model is intriguing for many reasons, the most obvious of which is the absence of a *σ_mem_* term; it is simply too small to play a detectable role in most cases. The second point of note is that the value of *K_s_* in Equation (20) depends not on ζ but on ζ + *ΞV_m_*, showing how the capacitive coupling outlined in previous sections results in another measurable change in a surface parameter. However, the most intriguing part is in Equation (19), where the conductance equals the medium conductivity divided by the radius (suggesting conduction around the particle through the bulk medium), less that of the surface conduction value. This suggests that the capacitive coupling actively reduces surface conduction, for example by reducing the number, availability, or polarity of charge carriers in the double layer.

Another intriguing aspect of this is that whilst the DEP-derived values of *G_spec_* are large for cells that are typically in suspension in the body (blood cells, macrophages, etc.), they are generally small-to-zero for cells found in tissues. Furthermore, the suspension cells often have relatively depolarized values of *V_m_* (typically at −20 mV or less), whereas adherent cells often have more polarized values (nearer the oft-quoted −70 mV). It is possible that the reason for this might be the active suppression of surface conduction, or rather of the conditions which are generated by the mechanisms responsible for surface conduction, such as manipulation of the anion/cation balance immediately outside the cell.

### 4.5. The Low-Frequency Dispersion in the Clausius–Mossotti Factor Yields Patch-Clamp-Compatible Parameters

**Figure 7:**
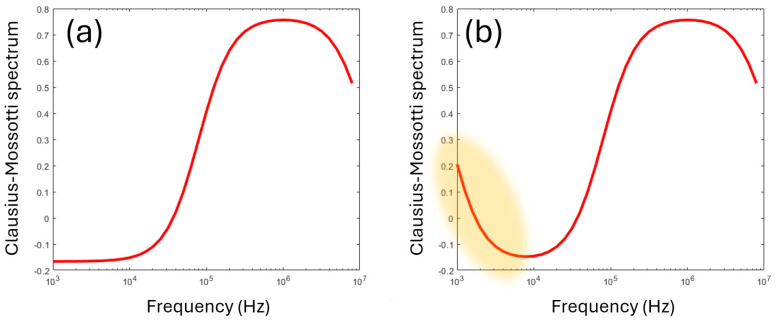
(**a**) The standard Clausius–Mossotti model exhibits a plateau at low frequencies (below 10^4^ Hz in the figure). However, experimental data usually exhibit a lift in polarizability resembling an additional dispersion, highlighted in (**b**) in orange.

The final component of the Clausius–Mossotti spectrum is arguably no such thing; it is an anomalous effect that appears in DEP spectra, particularly those extending to frequencies below 10 kHz. In this frequency range, DEP spectra often exhibit an upswing in polarizability, creating an additional very-low-frequency dispersion (typically below 10 kHz) shown schematically in Figure 7. This is not strictly a Clausius–Mossotti phenomenon—it is not based on interfacial polarization—but does appear as a component of DEP spectra taken and is commonly referred to as part of the “Clausius–Mossotti spectrum”.

Analysis of the RBC data set, as well as HeLa, neurons, chondrocytes, THP-1 cells, and platelets suggested that the effect is due to the polarization of the electrical double layer itself. This effect has been previously observed in low-frequency polarization in the DEP spectra of nanoparticles at high conductivity [70]. This additional polarization term was found to resemble a classical resistor–capacitor single-order filter characterized by a time constant, in line with observations of double layer polarization made by other methods [44].

**Figure 8:**
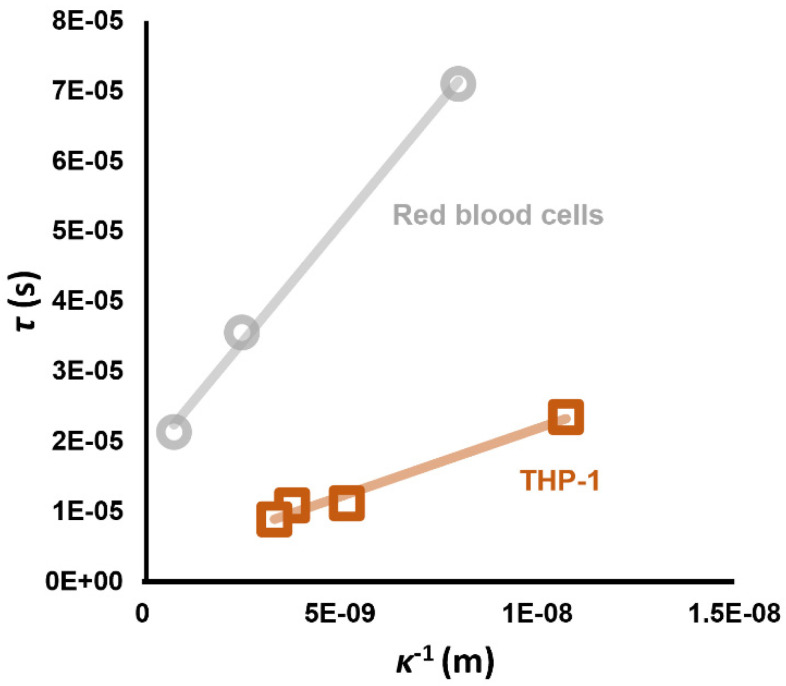
Linear relationships have been observed between the time constant of the low frequency dispersion, and the calculated Debye length κ^−1^ at the value of *σ_med_* at which the measurement was taken. The y-intercept value is comparable to values of τ measured using patch clamp, for the few cell types for which this parameter has been published. Data from Hughes et al. [68].

The low-frequency dispersion (often referred to as the low frequency “tick”) is an additional term which acts like a low-pass filter added to the standard C-M model (Equation (4)), thus:

(21) $$F_{DEP}\propto Re\left[\frac{\varepsilon_{cell}^{*}-\varepsilon_{med}^{*}}{\varepsilon_{cell}^{*}+2\varepsilon_{med}^{*}}\right]+\frac{a}{1+j\omega \tau }$$

where *τ* is the time constant and *a* is the amplitude. When *τ* was examined, it was found to exhibit a highly linear relationship to the double layer length 1/*κ* (examples of which are shown in Figure 8) which varies with medium conductivity when observed in RBCs under the range of conditions described previously, plus THP-1 monocytes before and after treatment with potassium channel blocker TEA, as well as primary neurons and HeLa cervical cancer cells.

Earlier work using homogeneous nanoparticles [70] suggested that this low-frequency effect arises from the polarization of the double layer; this work suggested that it in fact represented the polarization of a combination of the electrical double layer and the cell membrane. If one considers the single-order filter as arising from the capacitance and conductance of these layers, forming a filter with the classical time constant as follows:

(22) τ=C/G

then we can fit this to the linear data produced for the different cell types. As straight lines, these are characterized by their slopes and y-intercepts.

Since *τ* is proportional to double layer length 1/*κ*, we can examine these two components separately. The capacitance of the electrical double layer is usually calculated via the linearized Poisson–Boltzmann expression [44]:

(23) $$C=\varepsilon_{0}\varepsilon_{dl}\kappa$$

where *ε_dl_* is the permittivity of the double layer. It can be seen from Equation (8) that *κ* is related to the square root of medium ion concentration *c*, and hence in turn to the medium conductivity. The *G* term was found to fit best when Equation (19) was used. Since (from Equation (23)) capacitance *C* is proportional to *κ* and hence to c^0.5^ and thus to *σ_med_*^0.5^, and as *G_spec_* is proportional to *σ_med_* (in Equation (19)), then from Equation (22) we observe that *τ* would indeed be proportional to *σ_med_*^−0.5^ and hence 1/*κ*.

If we examine Equation (19), we find that *G_spec_* is described by the sum of two components; the first (*σ_med_*/2*r*) contains no variables relating to the cell other than the cell radius *r_cell_*. We can examine the contribution of this component to the Tick effect by calculating Equation (22) using Equation (19) to calculate *G_spec_* and setting ζ to 0 mV. A graph of *τ* vs. *κ*^−1^ shows a linear relationship, with a slope that is very similar to that observed for cells with a low value of *V_m_*, such as the RBCs in Figure 8. Where cells had more polarized *V_m_*, the second component deriving from Equation (20) acts to decrease the gradient, with THP-1 cells (*V_m_* ≈ −25 mV), having a lower gradient, and neurons (*V_m_* ≈ −70 mV) being even lower [68]. Modeling of the effect using this set of equations was found to accurately predict the time constant when Equation (13) was used to first predict *V_m_* from the standard Clausius–Mossotti model, and then using this to calculate *τ*.

Another key feature of the relationship between *τ* and *κ*^−1^ is the *y*-intercept; that is, the predicted value of *τ* when *κ*^−1^ is 0 nm. Examination of this suggests that, if a standard value of membrane capacitance of 9 mFm^−2^ (the capacitance of a flat membrane) [62] is used, then the whole-cell resistance value *R_wc_* can be obtained from the estimated value of *τ* for *κ*^−1^ = 0. This, combined with the *C_wc_* values which have already been demonstrated using conventional DEP modeling [32,33], means that all of the resting electrical parameters associated with patch clamp and other canonical electrophysiology measures have been identified.

The amplitude of the low-frequency dispersion (parameter *a* in Equation (21)) was observed to vary between cell types, and sometimes between conductivities, but was not numerically tied to any specific parameters; suggestions were made that it might relate to cell surface area, or eccentricity (the degree to which it is prolate or oblate rather than spherical), depending on whether the amplitude was compared to that of the CM factor itself, or taken as an absolute. Certainly, a measure of the latter might be a useful tool and additional parameter to extract, and as such may be worth future pursuit.

### 4.6. A Note on Capacitance

Capacitance has not been studied in the same depth as the conductivity- and potential-related parameters, but it has been observed to change with double layer length in RBCs [16,49,69]; the model for low-frequency dispersion also used the value based on the double-layer capacitance from Equation (23). However, it is also known that capacitance can vary between cell types at the same conductivity and indicates membrane folding (e.g., [13]). We also know that, at low conductivities, conventional DEP modeling yields values similar to those observed by other methods [61]. A useful model may be a series combination of the two, which would be similar to the (much lower) membrane value at low medium conductivity; this fits the RBC models presented here well but needs to be explored across other models before a definitive report can be made.

## 5. Discussion

Electrophysiology as a science has fascinated scientists for decades, and yielded Nobel prizes for several of them (including Erwin Neher, Bert Sakmann, Alan Hodgkin, John Eccles, and Andrew Huxley). However, despite DEP being touted as an electrophysiology tool for several decades, it has never gained traction with the wider electrophysiology community despite offering relatively easy access to several passive electrophysiological parameters. This cannot simply be attributed to the electrophysiology community being too conservative to adopt new methods; beyond patch clamp, there are many other methods in regular use, including voltage-sensitive fluorescence markers, pH changes, or rubidium radioactivity [71]. Given this diversity of methods, and the absence of DEP, we must instead look to the shortcomings of our method; if we are not heard, it is because we do not speak the language. Whilst DEP has been used to study the differences in biology between cells, or study the effects of interventions on cells, it has done so in a way that is not meaningful to most electrophysiologists. This is even the case where the distance is small; parameters such as *G_spec_* and *C_spec_* have been referred to as “those DEP parameters” by scientists who routinely use *C_wc_* and *R_wc_*, despite the two being readily convertible by multiplying by the presumed cell surface area. If DEP is to be taken seriously beyond its practitioner base, it is necessary to learn the language and present results in a more widely interpretable way. This is beginning to happen (e.g., [33]) but is still not widespread. The principal issue with the broader adoption of DEP has been its inability to reproduce some of the electrophysiological parameters that standard methods have used for decades—in particular *V_m_*, and to a lesser degree values of *R_wc_* in line with those observed by patch clamp; these simply do not appear on the Clausius–Mossotti spectrum in any meaningful way, though links have previously been drawn between cytoplasm conductivity and membrane potential [66].

**Figure 9:**
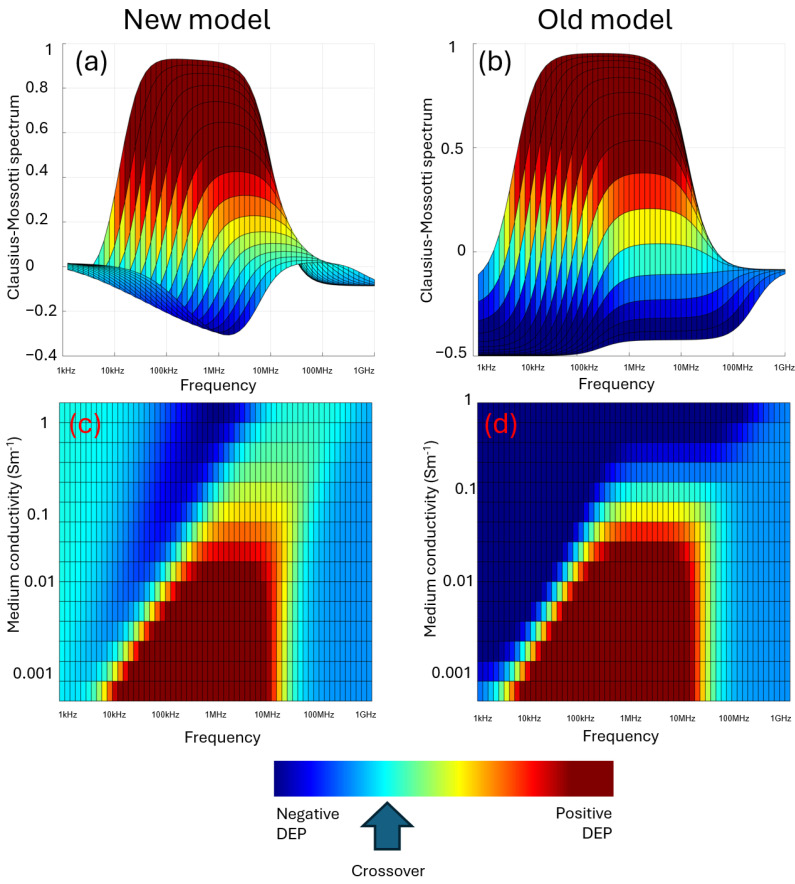
Three-dimensional plots of the Clausius–Mossotti factor as a function of both frequency and medium conductivity. When examined as overlapping Clausius–Mossotti responses, the new model (**a**) exhibits less negative DEP at both low frequencies (due to the low-frequency polarization effect) and high frequencies (due to the measured value of *σ_cyto_* being related to extracellular *σ_med_*) than the standard Clausius–Mossotti model (**b**). When plotted against both *σ_med_* and frequency, the new model (**c**) exhibits positive DEP over a shorter range of frequencies at low conductivity than the old model (**d**). An important difference visible in these plots is that the old model predicts that at high *σ_med_*, cells only exhibit negative DEP; the new model exhibits positive DEP even at high conductivity, though with reduced magnitude and occurring at higher frequencies.

**Figure 10:**
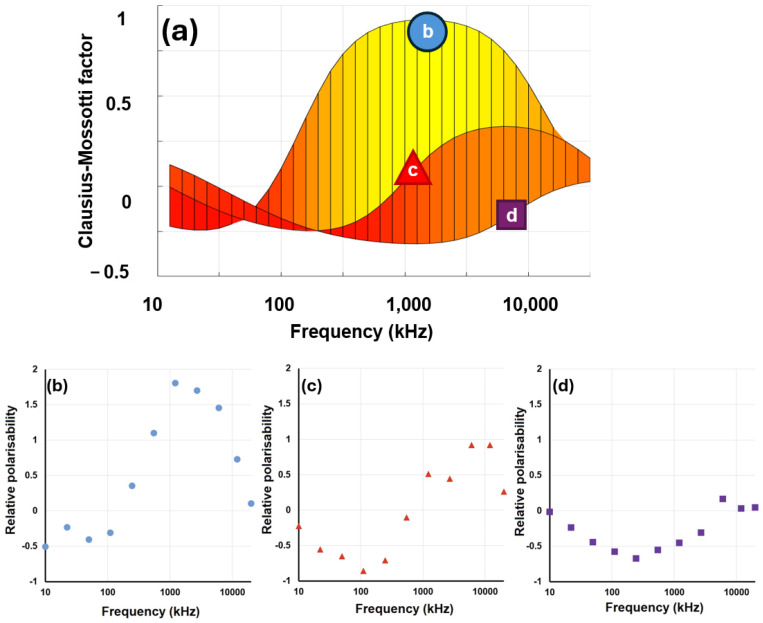
(**a**) The predicted DEP response of red blood cells (RBCs) across three conductivities, determined using all of the relationships presented in this paper. The lines on panel (**a**) can be compared to experimental data from RBCs taken at the same medium conductivities: (**b**) 16 mS^−1^, (**c**) 160 mS^−1^, (**d**) 1.6 S^−1^. Note the *y*-axes of plots (**b**–**d**) indicate outputs from the 3DEP, not the Clausius–Mossotti factor itself (to which they are related, by an arbitrary scaling factor).

However, it has emerged through the works covered here that the Clausius–Mossotti factor is not the two-dimensional graph we have assumed it to be, with DEP response mapped against frequency alone, as shown in Figure 2b or extended to Figure 7. Instead, we should consider it as a three-dimensional plot such as in Figure 9, where DEP response is plotted against both frequency and medium conductivity. This provides a plot that, as well as showing the landmarks of initial and final value and the two dispersion frequencies yielding the usual parameters of *G_spec_*, *C_spec_*, and *σ_cyto_* (and occasionally *ε_cyto_*), also identifies gradients in the low-frequency dispersion frequency, *G_spec_* and *σ_cyto_*, that give the parameters of *R_wc_*, ζ-potential, *Ξ,* and most importantly *V_m_*. Since these relationships are generally linear, two conductivities may be sufficient, though there is benefit in studying more in order to assess reproducibility. Furthermore, since the parameters vary linearly across a wide range of *σ_med_* values (we have investigated this over three orders of magnitude), it is only necessary to study a relatively small conductivity window to predict the response elsewhere. Empirically, we have found that the 150–250 mSm^−1^ range works well; below 50 mSm^−1^ it becomes harder to accurately maintain a measurable solution conductivity against factors such as resuspension carryover, whilst above 250 mSm^−1^ some highly conductive cells have high-frequency dispersions beyond the range of many signal generators (which commonly go to 20 MHz). This is however a rule of thumb and is not intended to discourage exploration beyond these limits. For example, the RBC spectra published by Hughes et al. in 2021 [16] can be accurately modeled across two orders of magnitude in *σ_med_*, as shown in Figure 10, by using Equations (2)–(4) adapted with Equation (21) to include the low-frequency dispersion; Equations (18)–(20) to model *G_spec_*, using values of *V_m_* derived from *σ_cyto_* using Equation (13), to calculate *σ_mem_*; using this value of *G_spec_* together with Equations (22) and (23) to model the low-frequency dispersion; and using a series model of membrane capacitance and double-layer capacitance determined from Equation (23) to determine *C_spec_* and hence *ε_mem_*. As can be seen, this yields highly representative curves of DEP behavior across a wide range of both frequencies and conductivities.

It is also worth noting that the change in our assumptions about the DEP response may have implications for the design of DEP apparatus, in particular for DEP separators. The revised model predicts much lower negative DEP forces than had previously been predicted, as evidenced by the lack of significant negative DEP response in Figure 9. Whilst this does not deny that negative DEP response exists and has been successfully used for DEP separation for decades, it does potentially recalibrate how we design DEP separators. In particular, it means that separators based on a strong negative DEP response might require stronger field gradients than those principally exploiting positive DEP. It is also worth noting that negative DEP exhibits a minimum just below the frequency of the first dispersion, rather than being similar from that point down to DC, suggesting that precise tuning of separation frequency may be required.

The model presented here is incomplete; there are some parameters which have not been fully explored. For example the amplitude of the low-frequency dispersion has been described qualitatively in terms of its likely origin, but there is no expression yet that might yield useful physiological data; the relationship between *C_spec_* and the actual membrane capacitance has yet to be studied in detail, though the way in which it varies does align with predictions from Equation (23). The parameter *ε_cyto_* remains beyond the reach of many generators and it is not known if this varies with medium composition. Other questions arise from the way in which we construct our models and what it tells us about the electrical structure of the cell and associated double layers. Of particular note is that *G_spec_* appears to be the result of summing the membrane and double layer conductances, a model that dates back to Arnold and Zimmermann [60]. There is also evidence that the capacitances of membrane and double layer are summed [16,64,69]. However, any high school physics student knows that capacitances and conductances only sum when in parallel; when in series, the reciprocal is given by the sum of the reciprocals of the components. This suggests that these components act tangentially—through the double layers parallel to the field lines—more than they do from cell exterior to interior. Finally, the offset values *Ψ_offset_* used for determination of *V_m_* in different conductivities are as yet determined only empirically. Since we have values that appear to work across all cell types, this does not need to be understood in order to make the model work; instead, it warrants investigation to understand what this tells us about the nature of *V_m_* itself.

Indeed, there are many aspects of the model presented here that raise questions about the nature of *V_m_*. *V_m_* as described by Equation (6) is of a system out of equilibrium, where ion channels are used to constantly pump ions against their concentration gradients to maintain the diffusion gradient giving rise to *V_m_*. However, the fact that *σ_cyto_*, which depends on the cytoplasm in concentration, varies linearly with *σ_med_* suggests that the process might be more of an equilibrium. One model that describes this is the Gibbs–Donnan model of permeant and non-permeant ions [36]; this is often dismissed for higher values of *V_m_* as the required cytoplasm ion concentration would drive so much water ingress due to osmosis that the cell would burst. However, the model presented here suggests that this excess charge might only be present in the electrical double layer, and that it is this value that we actually observe when we extract the *σ_cyto_* parameter using CM Modeling. This would change our understanding of the origin of *V_m_*, at least for non-excitable cells; an issue with these models is that all were developed for excitable cells (muscle, nerve) between the 1930s and 1950s [39,40,41,42,43] and subsequently applied to all cells. These results suggest that this might not be the best approach. The work also highlights the importance of capacitance in the formation of *V_m_* and may point towards new models of cell electrical behavior—and an expanding of our understanding of the role of the cellular electrome in wider cell biology

## 6. Conclusions

The Clausius–Mossotti spectrum has been used for the determination of cellular electrical properties for three decades. The ability it confers to allow dielectrophoresis to determine passive electrical properties of cytoplasm and membrane have allowed DEP to become a functional tool for cell electrophysiology. However, the parameters yielded by this method have found little traction with classical electrophysiology. Recent work has demonstrated that these parameters—and importantly, the way in which they change in media of different conductivity—allow the unlocking of further parameters that form a “Rosetta stone” between DEP, surface science, and conventional electrophysiology, and potentially unlocks new understandings of the role the cellular electrome plays in the function of the cell.

## Figures and Tables

**Figure A1:**
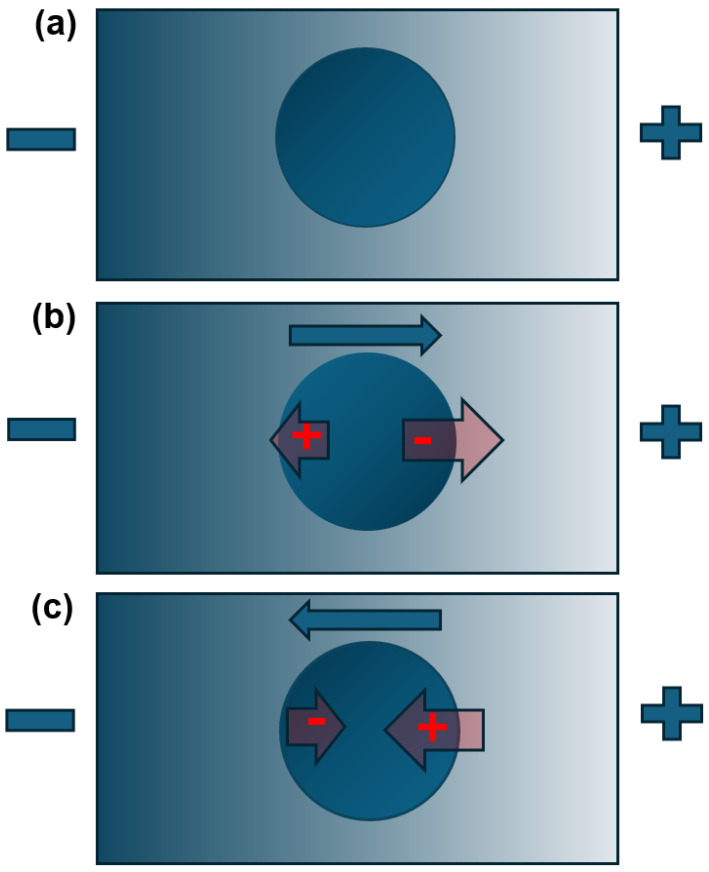
Consider three particles suspended in a nonuniform electric field (brighter = higher magnitude). At the instant of measurement, the right side is positively charged and left side negatively charged, though if this is an AC field, these polarities will vary from one instant to the next. The first particle (**a**) is unpolarized and does not interact with the field. The second (**b**) is more polarizable than the suspending medium, leading to a dipole orientation where the signs interact to create attractive forces either side of the dipole. Since the force on the side with highest field will dominate, the particle moves in the direction of increasing field magnitude (positive DEP). If the particle is less polarizable than the medium (**c**), the dipole will orient in the opposite direction, leading to repulsive electrical interactions. Again, the side with the highest electric field dominates and the particle is repelled down the field gradient (negative DEP).

**Figure A2:**
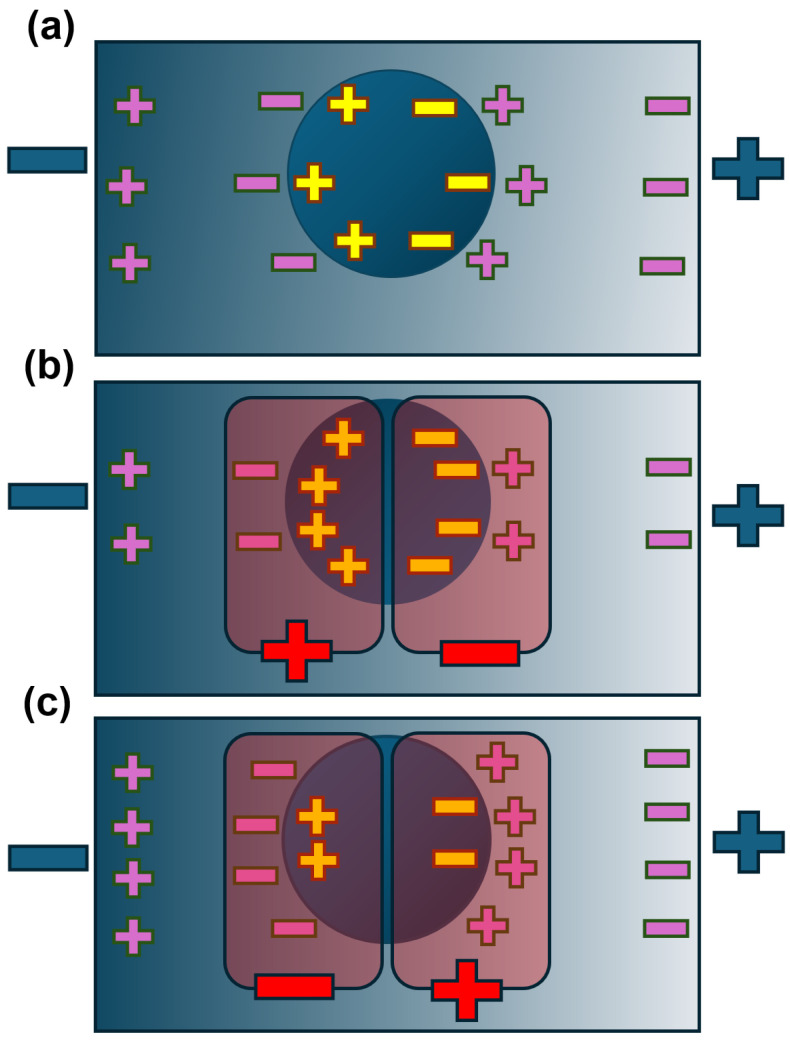
The charge distributions due to the imposed electric field gradient in Figure A1a. The figures assume that the particle and medium are made of materials with different dielectric properties (conductivity and permittivity). At some frequencies, the polarization of the particle (represented by the yellow charges) equals the polarization of the medium (purple charges); the net charge at either pole is zero (**a**). If the particle is more polarizable than the medium at the friequency of the applied field (**b**), this will lead to an increase in the charge on the particle compared to the medium; if we examine the interfaces between particle and medium, the net charge (red) at each pole is oriented as the particle in Figure A1b, and it will experience positive DEP. If, at a different frequency, the medium is more polarizable than the particle (**c**), then the net charge on both sides is reversed, giving negative DEP as seen in Figure A1c. Note that although the *net* dipole (yellow) changes direction, the individual dipoles of particle and medium *do not change direction*—they only change in relative magnitude.

## Appendix A. DEP Basics

Any charge experiencing an electric field will experience a force, which will induce it to move. This motion is called *electrophoresis* and forms the basis of many phenomena, including providing the basis of molecular separation in the widely used molecular biological technique of *gel electrophoresis*. In gel electrophoresis, charged molecules are suspended in a uniform (same everywhere), DC (“direct current”; that is, the voltage is constant and unvarying) electric field **E**, where they experience a force **F** in accordance with Coulomb’s law:

**F** = *q***E** (A1)

where *q* is the molecular charge.

If the particle is uncharged but a dielectric (that is, one with capacitance), when suspended in an electric field it will be subject to a redistribution of charges in response, a process called *polarization* due to the formation of electrical “poles” along the axis of the electric field lines. Each of these poles will interact with the field, producing equal and opposite forces which cancel out, so that the particle does not move. However, if the field is *non*-uniform (that is, stronger in some places and weaker in others), then the forces will not be equal; the force on the side where the field is greatest will dominate over that at the weaker end, leading to net movement of the particle. This is *dielectrophoresis* (DEP), as illustrated in Figure A1.

DEP differs from electrophoresis in two important ways. Firstly, the motion is dependent on an electric field gradient, not just the presence of an electric field. Secondly, because it is the gradient that defines the direction rather than the absolute value—or even direction—of the electric field, it is possible to use alternating current (AC) electric fields instead of DC fields; if the polarity of the imposed field flips, the orientation of the induced dipole also flips and the direction of the force remains the same. This is important, because unlike fixed charges, the magnitude or even the direction of an induced dipole can vary as a function of frequency. Furthermore, a charged dielectric particle will experience both DEP and electrophoresis, but if the field constantly alters direction, then the average displacement due to electrophoresis averages to zero and only DEP remains. DEP acts along the direction of increasing field gradient; if going up the field gradient (in the direction of highest electric field), it is called positive DEP (sometimes styled pDEP or +DEP), shown in Figure A1b; if going in the opposite direction, negative DEP (nDEP, −DEP), as shown in Figure A1c.

Whether the particle experiences positive or negative DEP (and the magnitude of force experienced) is defined by the relationship between the polarizability of the particle at the applied frequency, relative to the polarizability of surrounding medium. Furthermore, the same particle may experience positive DEP at some frequencies, and negative DEP or no force at all at others. This is shown schematically in Figure A2. The relationship that describes the relative polarizability of the particle with respect to the suspending medium is given by the Clausius–Mossotti factor, the subject of this paper.

## Appendix B. C–M Factor for Ellipsoidal Particles

The standard embodiment of the Clausius–Mossotti factor described above is based on the assumption that the cell is, or can be approximated to be, a sphere. This generally holds; for example, even when oblate red blood cells are Modeled as spheres, the resulting value of *C_spec_* can be converted to a whole-cell value similar to that obtained by patch clamp, simply by multiplying *C_spec_* by the surface area of the sphere that was used in the model. Ellipsoidal versions of the Clausius–Mossotti factor do exist, but are not widely used as they require knowledge of both major and minor axis radii, whereas spherical cells are easy to measure using either image analysis software or automated cell counters— reliably measuring two axes is difficult. Nevertheless, the theory is presented here for cases where dimensions (such as the length and radius of a bacterium) are known. The DEP force on an ellipsoid aligned with the field is given by [71,72,73]:

(A2) $$F_{\mathrm{DEP}}=\frac{2\pi abc}{3}\varepsilon_{m}Re\left[X(\omega )\right]\nabla E^{2}$$

where

(A3) $$X(\omega )=\frac{\varepsilon_{p}^{*}-\varepsilon_{m}^{*}}{\left(\varepsilon_{p}^{*}-\varepsilon_{m}^{*}\right)A_{\alpha }+\varepsilon_{m}^{*}}$$

and where α represents either the *x*, *y*, or *z* axis, and *A* is the depolarization factor along each axis (not the conductivity gradient, as earlier!), given by:

(A4) $$A_{x}=\frac{abc}{2}\int_{0}^{\infty }\frac{ds}{(s+a^{2})\sqrt{\left(s+a^{2})(s+b^{2})(s+c^{2}\right)}}\;A_{y}=\frac{abc}{2}\int_{0}^{\infty }\frac{ds}{(s+b^{2})\sqrt{\left(s+a^{2})(s+b^{2})(s+c^{2}\right)}}\;A_{z}=\frac{abc}{2}\int_{0}^{\infty }\frac{ds}{(s+c^{2})\sqrt{\left(s+a^{2})(s+b^{2})(s+c^{2}\right)}}$$

where s is the variable of integration. The polarization factors are interrelated such that *A*_x_ + *A*_y_ + *A*_z_ = 1.

For a prolate ellipsoid (*a* > *b*, *b* = *c*) such as a bacterium, this becomes:

(A5) $$F_{\mathrm{DEP}}=\frac{2\pi abc}{3}\varepsilon_{m}Re\left[\frac{\varepsilon_{p}^{*}-\varepsilon_{m}^{*}}{1+\left(\frac{\varepsilon_{p}^{*}-\varepsilon_{m}^{*}}{\varepsilon_{m}^{*}}\right)A}\right]\nabla E^{2}$$

where *A* is given by the expansion:

(A6) $$A=\frac{1}{3\gamma^{-2}}\left[1+\frac{3}{5}(1-\gamma^{-2})+\frac{3}{7}{\left(1-\gamma^{-2}\right)}^{2}+\ldots \right]$$

and where *γ* = a/b. Note that where the object under consideration is a sphere, *γ* = 1 and *A* = ^1^/_3_. In this instance, the equation can be reordered to produce the expression for the DEP force on a sphere, with the real term becoming the classical Clausius–Mossotti expression for a sphere. As with spherical cells, it is also possible to construct multi-shell models; the process for extending spheroids is similar to that shown for spheres, but with the real term being replaced by:

(A7) $$\varepsilon_{(i)eff}^{*}=\varepsilon_{i}^{*}\frac{\varepsilon_{i}^{*}+(\varepsilon_{(i+1)eff}^{*}-\varepsilon_{i}^{*})[A_{i\alpha }+v_{i}(1-A_{(i-1)\alpha })]}{\varepsilon_{i}^{*}+(\varepsilon_{(i+1)eff}^{*}-\varepsilon_{i}^{*})(A_{i\alpha }+v_{i}A_{(i-1)\alpha })}$$

where

(A8) $$v_{i}=\frac{a_{i}b_{i}c_{i}}{a_{i-1}a_{i-1}a_{i-1}}$$
